# Characterizing the supragingival microbiome of healthy pregnant women

**DOI:** 10.3389/fcimb.2022.1016523

**Published:** 2022-11-17

**Authors:** Yangyang Zhang, Zeyu Wu, Ling Li, Xiaohe Wang, Wenxian Fan, Jin Zhao

**Affiliations:** ^1^ Department of Cariology and Endodontics, The First Affiliated Hospital of Xinjiang Medical University (The Affiliated Stomatology Hospital of Xinjiang Medical University), Urumqi, China; ^2^ Stomatology Disease Institute of Xinjiang Uyghur Autonomous Region, Xinjiang Medical University, Urumqi, China; ^3^ Department of Obstetrics and Gynecology, The First Affiliated Hospital of Xinjiang Medical University, Urumqi, China

**Keywords:** supragingival plaque, diversity, 16S rRNA gene sequencing, pregnancy, functional pathway

## Abstract

The ecological characteristics and changes of the supragingival plaque microbial community during pregnancy are poorly understood. This study compared the microbial community characteristics of supragingival plaque in pregnant and non-pregnant women, with the aim of identifying specific microbial lineages and genera that may be associated with pregnancy. Thirty pregnant women were randomly selected from the First Affiliated Hospital of Xinjiang Medical University and divided into groups based on pregnancy trimester: first trimester (group P1, n=10, ≤12 weeks), second trimester (group P2, n=10, 13–27 weeks), and third trimester (group P3, n=10, 28–40 weeks). Ten healthy non-pregnant women (group N) were enrolled as the control group. Supragingival plaque samples of all subjects were collected and oral microbial composition was surveyed using a 16S rRNA gene sequencing approach. Statistical analysis was performed using a nonparametric test. The Chao 1 index of P3 was significantly lower compared with that of N, P1, and P2 (*P*<0.05). The Simpson indices of P2 and P3 were significantly higher than that of N (*P*<0.05). The Shannon index of P2 was significantly higher compared with that of N (*P*<0.05). Principal coordinate analysis (PCoA) showed different clustering according to the pregnancy status. Linear discriminant analysis effect size (LEfSe) revealed that the microbial species in group N that were significantly different from those of other groups were concentrated in the genus *Neisseria*. Species in P1 that were significantly different from those of other groups were concentrated in the genus *Tannerella*, while those in P2 and P3 were concentrated in the genus *Leptotrichia*. A total of 172 functional pathways were predicted for the bacterial communities in this study using PICRUSt2. Principal Component Analysis (PCA) showed that most predicted functional pathways clustered together in N and P1 and in P2 and P3. LEfSe analysis revealed that 11 pathways played a discriminatory role in the four groups. This work suggests a potential role of pregnancy in the formation of supragingival plaque microbiota and indicates that physiological changes during pregnancy may convert supragingival plaque into entities that could cause harm, which may be a risk factor for maternal health. Furthermore, findings from the study provide a basis for etiological studies of pregnancy-associated oral ecological disorders.

## Introduction

A broad microbiome consisting of bacteria, viruses, phages, and fungi is present in almost every part of the body (Proal et al., 2017), and the oral cavity is one of the five major habitats of human microbes (Human Microbiome Project, 2012). The oral microbiome is an essential part of the human microbiome (Human Microbiome Project, 2012) and the expanded Human Oral Microbiome Database (eHOMD) includes 775 oral microbial species (Escapa et al., 2018). Oral microbiota interact with the host microenvironment in a complex way to maintain the dynamic balance of oral microecology. Smoking (Al Bataineh et al., 2022), circadian rhythm disorders (Chellappa et al., 2022), poor dietary habits (Chen et al., 2022; Santonocito et al., 2022), obesity (Hudek Turkovic et al., 2022), hormones (Ye and Kapila, 2021), oral hygiene (Harding et al., 2017; Sedghi et al., 2021), and other factors can alter this balance, and imbalance of human oral microecology is closely related to local and systemic diseases of the oral cavity. This includes oral diseases such as caries (AlEraky et al., 2021; Pitts et al., 2021), periodontal disease (Chen et al., 2018; Usui et al., 2021), and oral cancer (McIlvanna et al., 2021; Stasiewicz and Karpiński, 2021) as well as systemic diseases such as diabetes (Zhang et al., 2021a), cardiovascular disease (Deraz et al., 2022), rheumatoid arthritis (Kroese et al., 2021), inflammatory bowel disease (Read et al., 2021), colorectal cancer (Yu et al., 2022), and premature birth (Vander Haar et al., 2022; Yang et al., 2022). Oral microbiology can therefore be used as an approach to explore the biomarkers of oral diseases and related systemic diseases. However, to characterize changes in the oral microbiome during disease, it is necessary to understand the species composition and functional genetic metabolic pathways of the oral microbiome in healthy populations.

Adverse pregnancy outcomes include preterm birth, stillbirth, low birth weight, pre-eclampsia, and so on, and affect more than 20% of newborns globally each year (Ye and Kapila, 2021). Adverse pregnancy outcomes can directly lead to infant/fetal death or congenital disabilities and may also be associated with the development of chronic diseases in adulthood, placing a heavy economic burden on families and society (Rogers and Velten, 2011; Chen et al., 2012). However, half of the causes of adverse pregnancy outcomes remain unknown (Ye and Kapila, 2021). Intrauterine infections play a major role in adverse pregnancy outcomes (Ye and Kapila, 2021). Intrauterine placental bacterial infection most likely originates from upstream infection by lower genital tract bacteria and bloodstream infection by oral bacteria (Han Y and Wang, 2013; Vornhagen et al., 2016). Women undergo changes in their oral microecological environment during pregnancy owing to hormonal and immunological alterations (Ye and Kapila, 2021). Such changes increase the susceptibility of pregnant women to oral diseases like periodontal disease and gingivitis (Saadaoui et al., 2021). Previous studies have indicated that periodontal disease is a significant risk factor for the development of adverse pregnancy outcomes (Daalderop et al., 2018; Bobetsis et al., 2020). Two different mechanisms have been proposed to explain how periodontal disease affects pregnancy outcome. The first mechanism is that oral microorganisms directly invade the fetoplacental unit (Saadaoui et al., 2021; Xu and Han, 2022), while the second mechanism is that oral microbes produce inflammatory mediators that affect the fetoplacental unit (Xu and Han, 2022). In both cases, microorganisms or inflammatory mediators entering the fetoplacental unit can cause an inflammatory response, which, in turn, can affect fetal development, lead to spontaneous abortion, or trigger preterm labor and delivery (Saadaoui et al., 2021). Therefore, studies of the oral microbiome during pregnancy are necessary to improve prediction and interventions of adverse pregnancy outcomes.

Current findings regarding the differences in oral microbial composition between pregnant and non-pregnant women and the dynamics of the oral microbiome during pregnancy are inconsistent. Numerous studies have reported significant differences in oral microbiome composition between pregnant and non-pregnant women (Fujiwara et al., 2017; Lin et al., 2018; Balan et al., 2021; Zhang et al., 2021b), but a few studies concluded that there are no differences in oral microbiome composition between pregnant and non-pregnant women (Machado et al., 2012). Furthermore, there are reports suggesting that the oral microbial composition of pregnant women changes significantly (Borgo et al., 2014; Fujiwara et al., 2017; Lin et al., 2018), while other studies indicate that the oral microbial composition of pregnant women remains stable (Carrillo-de-Albornoz et al., 2010; Bisanz et al., 2015; DiGiulio et al., 2015). Hence, the composition and structural transformation of the oral microbiota during pregnancy remains poorly understood.

In this study, high-throughput 16S rRNA gene sequencing was used to compare the microbial community characteristics of supragingival plaque in 30 pregnant and 10 non-pregnant women, with the aim of identifying specific microbial lineages and genera that may be associated with pregnancy. Functional pathways of the microbial communities were also predicted. These findings may provide a broader understanding of pregnancy-associated oral microecological dysbiosis.

## Materials and methods

### Ethics statement

This study was approved by the Research and Ethics Committee of the First Affiliated Hospital of Xinjiang Medical University, China (file no. K202203-27) and was conducted according to the Declaration of Helsinki. All participants were fully informed about the study and provided written consent to participate.

### Participants

Forty subjects were recruited from November 2020 to February 2021 [sample size was referenced from previous studies (Bik et al., 2010; Lin et al., 2018; Balan et al., 2021)] and comprised 30 healthy pregnant women recruited from the obstetrics department of the First Affiliated Hospital of Xinjiang Medical University (Xinjiang, China) and a control group of 10 healthy non-pregnant women (group N). The pregnant women were divided into three groups (10 subjects per group) based on pregnancy trimester: first trimester (group P1, ≤12 weeks), second trimester (group P2, 13–27 weeks), and third trimester (group P3, 28–40 weeks). The inclusion criteria for the pregnant group were: (1) 20–35 years of age; (2) 28 natural teeth; (3) good oral health; (4) <42 weeks of gestation; and (5) good systemic health. The recruitment criteria for non-pregnant women were: (1) 20–35 years of age; (2) 28 natural teeth; (3) good oral health; and (4) good systemic health. Pregnant and non-pregnant women who met one of the following criteria were excluded: (1) systemic disease; (2) clinically diagnosable untreated oral lesions; (3) use of antibiotics within the past 3 months; and (4) smoking or alcohol consumption habits.

Demographic information was obtained through a self-reported questionnaire. Referring to the standards in the WHO Basic Methods for Oral Health Surveys, 5th edition (World Health Organization, 2013), a comprehensive oral health examination was performed on each subject by a professionally trained dentist under natural light, and the number of caries, missing teeth, and fillings (DMFT), plaque index (PLI), and gingival index (GI) were recorded by an assistant. Dental caries were recorded according to WHO criteria (World Health Organization, 2013), and prevalence of caries was expressed by the number of decayed, missing, and filled teeth (DMFT index).

Oral hygiene status was assessed using the PLI. Patients rinsed their mouth with water, then used a cotton swab or small cotton ball dipped in 2% neutral red solution and applied it to the tooth surface near the gingival margin, then rinsed again and checked the stained area of the tooth surface. The PLI is recorded as six levels, and the scoring criteria are: 0=No plaque on the tooth surface; 1=Scattered spot plaque on the gingival margin of the tooth neck; 2=Wide band of continuous narrow plaque on the tooth neck not more than 1 mm; 3=Area covered by plaque on the tooth neck more than 1 mm, but less than 1/3 of the tooth surface; 4=Area covered by plaque at least 1/3 of the tooth surface, but not more than 2/3; 5=Area covered by plaque 2/3 of the tooth surface or more.

Gingival health status was assessed using the GI. A periodontal probe was placed in the gingival margin at the opening of the gingival sulcus and gently slid along the gingival margin, only slightly touching the gingival tissue. Four levels of gingival tissue are scored: 0=Healthy gums; 1=Mild gingival inflammation: gums with mild color change and edema, no bleeding on probing; 2=Moderate gingival inflammation: gums with red color, edema, and bleeding on probing; 3=Severe gingival inflammation: gums with marked redness, swelling or ulceration, and a tendency to bleed spontaneously. A score of 0 is normal gums, while 1, 2, and 3 correspond to mild, moderate, and severe gingivitis, respectively.

### Sample collection

Supragingival plaque samples were taken from six index teeth from each woman according to the methods recommended by the Manual of Procedures for Human Microbiome Project (Mcinnes PCMA, 2010). All subjects did not brush their teeth on the morning of the sampling and did not eat or rinse lightly for 2 hours prior to sampling. Sampling was performed by a professionally trained dentist in a simple dental chair used for oral examination. The six index teeth included two molars (#3 and #19), two premolars (#12 and #28), and two incisors (#9 and #25). The areas to be sampled were separated with a cotton roll and dried with a gentle stream of air from an air-water syringe. All supragingival plaque was removed from the surfaces of the selected index teeth using a Gracey spatula. The tip of the spatula was then dipped into 500 µL phosphate-buffered saline (PBS) in a 2-mL Eppendorf tube for 4–5 s and the face of the curette was wiped on the inside edge of the collection tube. The same procedure can be used immediately Sampling of the locus again. If a subject had little plaque, the arch counterparts to the index molar and premolar teeth were also be sampled. The lid of the Eppendorf tube was closed, and the tube was shaken for 4–5 seconds to maximize dispersion of the specimen in the fluid. Dental plaque samples collected from each participant were immediately frozen, transferred to the microbiology laboratory, and stored at −80°C until use.

### DNA extraction

Total bacterial genomic DNA was extracted using a PowerMax Soil DNA Isolation Kit (MOBIO, Carlsbad, CA, USA) following the manufacturer’s protocol. The concentration of DNA was measured using a NanoDrop2000 instrument (Thermo Fisher Scientific, USA).

### Deep amplicon sequencing

The V3–V4 region of bacterial 16S rRNA genes were amplified by polymerase chain reaction (PCR) using the primer pair 338F/806R. Reactions were performed in triplicate in a volume of 25.0 μL each. PCR conditions were an initial denaturation at 95°C for 5 min, followed by 25 cycles of denaturation at 95°C for 45 s, annealing at 55°C for 50 s, and extension at 72°C for 45 s, and then a final incubation at 72°C for 10 min. The amplification results were confirmed by 1% agarose gel electrophoresis. PCR amplicons were then individually purified using Agencourt AMPure XP (Beckman Coulter, USA) and pooled in equal amounts. Sequencing libraries were generated using the NEB Next Ultra II DNA Library Preparation Kit (New England Biolabs, USA) according to the manufacturer’s instructions. Illumina sequencing was performed by Beijing Allwegene Technology Co. Ltd. (Beijing, China) using the paired-end method and the Illumina MiSeq PE300 platform. Sequencing data have been uploaded to the NCBI (National Center for Biotechnology Information) sequence read archive (BioProject no. PRJNA826664).

### Bioinformatic analysis

The raw sequencing data were screened to remove sequences shorter than 230 bp and low-quality tags. According to the UCHIME algorithm process, all arrangements were then examined and assigned taxonomically with a 97.0% bootstrap truncation rate based on 16S rRNA gene combinations from the Human Oral Microbiome Database (HOMD, version 15.2). High-quality representative sequences were aligned and clustered into operational taxonomic units (OTUs) with a threshold similarity of 97.0%. The Ribosome Database Project (RDP) classification tool assigned all sequences to different taxonomic groups. The relative abundance of individual taxa within each community was estimated by comparing the number of sequences assigned to a particular taxon with the number of sequences obtained for that sample. The community structure was statistically analyzed at the taxonomic level of the phylum, genus, and species. The top clades and top genera were listed. Alpha-diversity analysis was performed using QIIME (v1.8.0), including Chao1, Simpson, and Shannon indices. For beta-diversity analysis, a heat map-based distance matrix using weighted uniFrac and unweighted uniFrac distances was employed. Differences in microbial community composition were analyzed using principal coordinate analysis (PCoA) (Lozupone et al., 2007). PCoA and bacterial taxonomic analysis were calculated and plotted in R v3.5.2 software. Microbial community composition was visualized using Sankey diagrams, which are flow diagrams in which the arrow width is proportional to the quantity (e.g. gene expression) to depict changes over time or hierarchy between nodes (Platzer et al., 2018). Linear discriminant analysis (LDA) effect size (LEfSe) was employed to identify the taxa most likely to explain the differences between groups. LEfSe uses a nonparametric Kruskal–Wallis rank sum test to assess different features with significantly different abundance between assigned taxa and performs LDA to estimate the effect size of each sequence variant, as reported by Segata et al. (2011). The LDA scores ranked the different taxa and are displayed on the LEfSe bars according to their effect sizes. For LEfSe analysis, data were first converted to log10 before the non-parametric Kruskal–Wallis rank sum test. A significant alpha level of 0.05 and an effect size threshold of three times larger differences were used to display the results in this study. Co-occurrence networks of the 50 most abundant genera were analyzed using Mothur and visualized *via* Gephi (https://gephi.org/). Spearman’s correlation coefficients were also calculated. Network edges were set using genera for which the ρ-value was >0.6 and significant (*P*<0.05). To predict metabolic pathways, a phylogenetic survey of the community was performed from the sequencing data by reconstructing the unobserved state (PICRUSt2), as shown previously (Douglas et al., 2020). Fastq sequence files for each sample were processed using QIIME2. Representative sequences were used as input files for the PICRUSt2 analysis pipeline. Metabolic pathways were assigned based on the Kyoto Encyclopedia of Genes and Genomes (KEGG) Ortholog (KO) database. Read abundance data for all predicted pathways were converted to relative abundance and subjected to LEfSe analysis by the Galaxy server (https://huttenhower.sph.harvard.edu/galaxy/) using an LDA score of 3.0 as the threshold level to identify pathway taxa most likely to explain intergroup differences.

### Statistical analysis

Results were expressed as median and interquartile range. The Kruskal–Wallis test was used to compare differences in general clinical characteristics of study subjects between groups and differences in oral microbial communities. SPSS (version 27.0) software was used for statistical analysis. Results were considered statistically significant when the probability value (*P*) was <0.05.

## Results

### Clinical characteristics of participants

A total of 40 women—30 pregnant women and 10 non-pregnant women–were recruited for this study. The characteristics of the study population are illustrated in **Table 1** and **Table S1** in **Additional File 1**. No statistical differences were found between the groups regarding age, race, and oral status characteristics (Kruskal–Wallis test, *P*>0.05). However, body mass index was higher in the second- and third-trimester groups compared with that in the non-pregnant control group (Kruskal–Wallis test, *P*<0.001).

**Table 1:** Demographic, medical, and oral status characteristics of study subjects.

Categories		Pregnant (n=30)			Non-Pregnant (n=10)	*P*-value
		1st trimester (n=10)	2nd trimester (n=10)	3rd trimester (n=10)		
Age (years)		28.50 (32.00, 25.75)	29.50 (32.25, 26.50)	28.50 (32.25, 27.75)	27.50 (33.00, 24.75)	0.90
Ethnicity	Han nationality	9	10	9	9	0.79
	Minority nationalities	1	0	1	1	
Education	<High school completion	6	5	3	4	0.58
	≥High school completion	4	5	7	6	
BMI		23.17 (24.17, 21.36)	23.43 (24.25, 23.22)	24.36 (24.52, 22.84)	21.38 (21.76, 20.60)	<0.001
Smoking status	Never smoked	9	10	10	9	0.56
	Former smoker	1	0	0	1	
	Current smoker	0	0	0	0	
Alcohol consumption	Abstainer	10	9	9	9	0.79
	Non-current	0	1	1	1	
	Current	0	0	0	0	
Sweet consumption	Once/day	9	8	7	9	0.75
	more than once/day	1	2	3	1	
	None	10	10	9	10	
Tooth brushing	Twice/day	9	9	10	9	0.79
	Once/day	1	1	0	1	
	Less than once/day	0	0	0	0	
Flossing habit	Once or more/day	1	3	2	3	0.67
	Less than once/day	9	7	8	7	
DMFT		1.00 (2.00, 0.75)	2.00 (2.00, 0.75)	1.00 (2.25, 0.75)	0.50 (2.00, 0.00)	0.59
PLI		1.00 (1.25, 1.00)	1.00 (2.00, 0.00)	1.00 (1.25, 0.00)	1.00 (2.00, 1.00)	0.81
GI		1.00 (1.00, 0.00)	0.00 (1.00, 0.00)	0.00 (1.00, 0.00)	1.00 (1.00, 0.00)	0.67

BMI, body mass index (kg/m^2^); DMFT, decayed, missing, filled teeth number; PLI, plaque index; GI, gingival index.

### Bacterial diversity of supragingival microbiota

**Figure 1:**
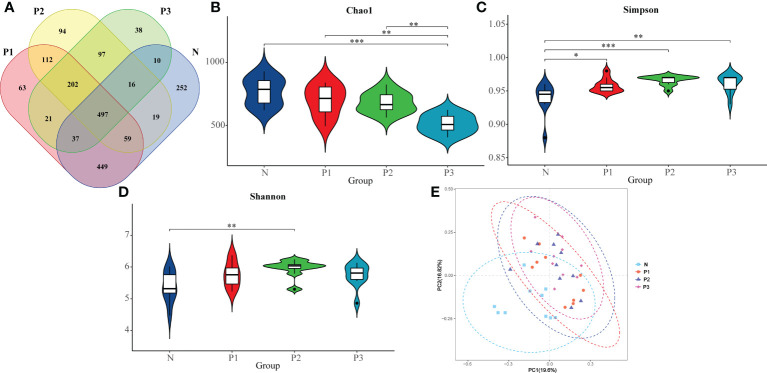
**(A)** Differences in number of OTUs among microbiota from N, P1, P2, and P3. **(B)** The comparison of Chao 1 index in N, P1, P2, and P3. **(C)** The comparison of Simpson index in N, P1, P2, and P3. **(D)** The comparison of Shannon index in N, P1, P2, and P3. **(E)** PCoA analysis revealing the bacterial communities in N, P1, P2, and P3. * P<0.05, ** P<0.01, *** P<0.001.

Illumina MiSeq sequencing produced 2909243 raw sequences, and after pre-processing, 2531486 usable, high-quality reads, with an average of 63287 ± 29723 sequences per sample, remained in the dataset (**Additional File 1: Table S1, Additional File 2: Figure S1A**). The average sequence length ranged from 400 to 440 bp (**Additional File 1: Table S3, Additional File 2: Figure S1B**). After removing the low-credibility OTUs, the taxonomic assignment of the sequences resulted in the identification of a total of 1335 OTUs in the supragingival microbiota. The number of OTUs in N, P1, P2, and P3 groups were 1339, 1440, 1096, and 869, respectively (**Figure 1A**). At this sequencing depth, the Shannon–Wiener curves for all samples have reached a plateau (**Additional File 2: Figure S2**), indicating that this sequencing depth is sufficient to capture the full range of microbial diversity.

The Chao 1 index reflects community species richness, while the Simpson and Shannon indices reflect microbial diversity (Hill et al., 2003). Kruskal–Wallis nonparametric tests and pairwise comparisons were conducted for the alpha-diversity indices of N, P1, P2, and P3. From The Chao 1, Simpson, and Shannon indices of these four groups were significantly different (**Table 2**). After pairwise comparison, the Chao 1 index of P3 was lower compared with those of N, P1, and P2, and the differences were statistically significant (*P*<0.05), indicating that the species richness of the oral microbial community decreased during late pregnancy (**Additional File 1: Tables S4, S5** and **Figure 1B**). The Simpson indices of P2 and P3 were higher compared with those of N and P1, and the differences were statistically significant (*P*<0.05), indicating that the microbial diversity of the oral microbial community decreased in mid- and late pregnancy (**Additional File 1: Tables S4, S5** and **Figure 1C**). The Shannon index of P2 was higher compared with that of N, and the difference was statistically significant (*P*<0.05), indicating that the microbial diversity of the oral microbial community increased in the middle of pregnancy (**Additional File 1: Tables S4, S5** and **Figure 1D**). It can be concluded that pregnancy may lead to a decrease in species richness of the oral microbial community. However, the results of the two indicators reflecting microbial diversity were inconsistent, possibly influenced by species evenness.

**Table 2:** Alpha-diversity indices of N, P1, P2, and P3.

Group		Chao 1	Simpson	Shannon
N		786.22 (867.61, 664.54)	0.95 (0.95, 0.93)	5.32 (5.82, 5.10)
P1		714.14 (826.80, 578.63)	0.96 (0.96, 0.95)	5.76 (6.08, 5.40)
P2		665.75 (746.40, 618.13)	0.97 (0.97, 0.96)	6.04 (6.11, 5.91)
P3		506.12 (582.77, 448.90)	0.97 (0.97, 0.95)	5.81 (6.01, 5.58)
*P*-value	All groups	<0.001 (**)	0.002 (*)	0.023 (*)
	N-P1	1	0.283	0.714
	N-P2	1	0.001 (*)	0.012 (*)
	N-P3	<0.001 (**)	0.022 (*)	0.661
	P1-P2	1	0.583	0.770
	P1-P3	0.011 (*)	1	1
	P2-P3	0.026 (*)	1	0.829

Each value is the median and interquartile range. *indicates significant differences (P<0.05); **indicates extremely significant differences between groups (P<0.001).

A heatmap of beta-diversity index was used to measure the dissimilarity coefficient between samples. Weighted and unweighted UniFrac distances were used to indicate the close proximity of the bacterial communities in the four groups. A lower number represents greater similarity in bacterial microbiota between samples in the heatmap. The heatmaps (**Additional File 2: Figures S3A, B**) show that the communities of N and P1 are closer together, suggesting that the two groups are more similar, while the distance between the communities of N and P2 and P3 is larger, suggesting that the communities of N and P2 and P3 are more different. These findings suggest that the microbial community of supragingival plaque may have changed during mid- and late-pregnancy.

In addition, the differences in microbial community composition of N, P1, P2, and P3 were analyzed by PCoA (**Figure 1E**), and PC1 and PC2 accounted for 19.60% and 16.82% of the total variation, respectively. The PCoA plot revealed that the microbial community composition of N was different from those of the other three groups and the differences were statistically significant (PERMANOVA, pseudo-F=2.4218, *P*=0.001). This suggests that pregnancy may have an effect on the composition of oral microbial communities.

### Community structure of supragingival microbiota

**Figure 2:**
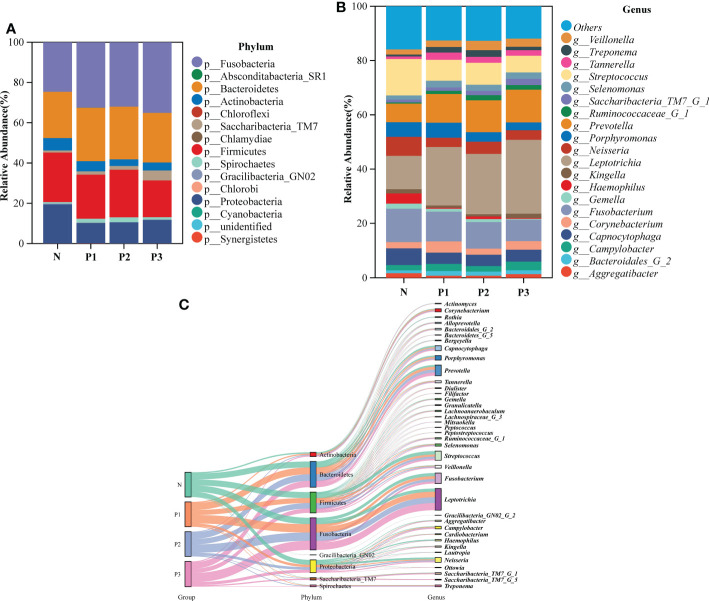
**(A)** The bacterial structure comparison in N, P1, P2, and P3 at the phylum level. **(B)** The bacterial structure comparison in the four groups at the genus level. **(C)** Sankey analysis of the bacterial structure comparison in the four groups at phylum and genus levels.

To characterize the bacterial distribution, the supragingival plaque microbiota of N, P1, P2, and P3 were analyzed in terms of relative taxonomic abundance. A total of 15 phyla, 32 classes, 53 orders, 98 families, 194 genera, and 503 species were detected. Bar graphs show the top 15 phyla in each of the four groups (**Additional File 1: Table S6** and **Figure 2A**). Four phyla—Fusobacteria, Bacteroidetes, Firmicutes, and Proteobacteria—were the most abundant, accounting for 91.56%, 91.07%, 92.18%, and 89.64% of the total OTUs in each of the N, P1, P2, and P3 groups, respectively. The top 20 genera in each of the four groups are depicted in bar charts (**Additional File 1: Table S7** and **Figure 2B**). *Leptotrichia* was the most abundant genus in the pregnancy group, followed by *Fusobacterium*, *Prevotella*, and *Streptococcus*, together accounting for 50.82%, 51.85%, and 53.22% in P1, P2, and P3, respectively. *Streptococcus* was the most abundant genus in the non-pregnancy group, followed by *Fusobacterium*, *Leptotrichia*, and *Neisseria*, which together accounted for 45.00% of the total OTUs in N group. Relative abundance at the phylum and genus levels could also be visualized by the Sankey diagram (**Figure 2C**), which showed results similar to those in **Figures 2A, B**. Collectively, these results suggest that the oral microbial community composition in the pregnant and non-pregnant groups was essentially the same at the phylum level but differed at the genus level.

**Figure 3:**
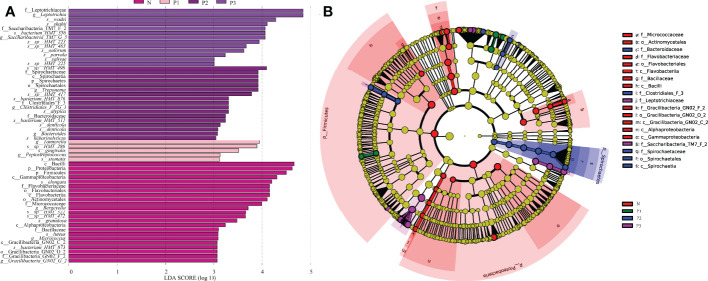
**(A)** Significantly enriched bacterial taxa in the different groups as determined by LEfSe analysis (LDA sore >3). **(B)** LEfSe taxonomic cladogram. The size of each node represents the relative abundance of the species. (p, phylum; c, class; o, order; f, family; g, genus; s, species).

The LEfSe method was used to identify the distinguishing phylotype that most likely explained the differences between N, P1, P2, and P3. LDA was combined with effect size measures for assessing the effect sizes of taxa (**Figure 3A**). A circular clade plot was created to show taxa with different abundances (**Figure 3B**). Species in group N that differed significantly from those of the other groups were concentrated in the genus *Neisseria*. In P1, the species that differed significantly from those of the other groups were concentrated in the genus *Tannerella*. In P2 and P3, the species that differed significantly from those of the other groups were concentrated in the genus *Leptotrichia*. (**Additional File 1: Table S8** and **Figures 3A, B**).

**Figure 4:**
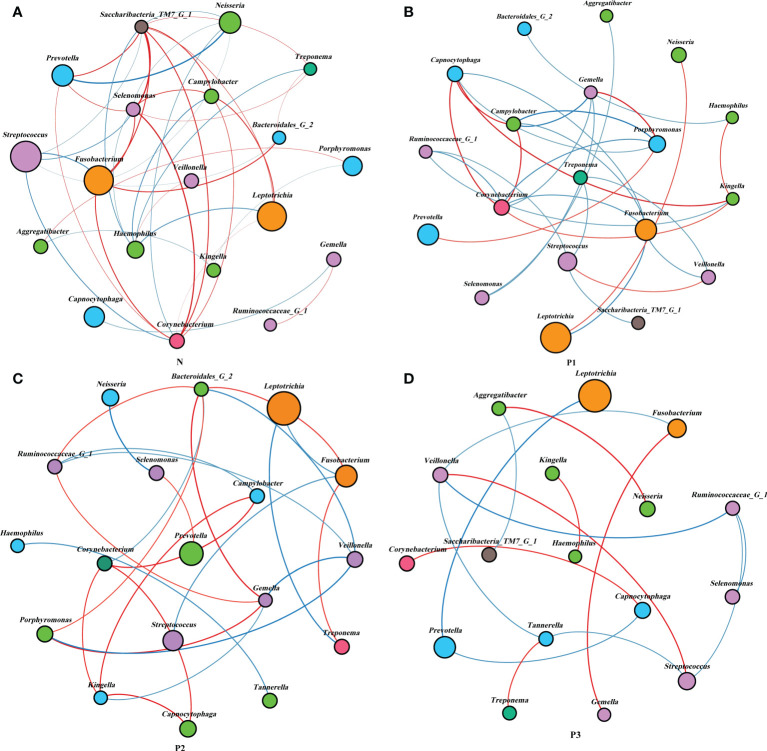
Network analysis between bacterial taxa in the four groups. Network analysis revealed the 20 most abundant genera (|SpearmanCoef>0.6 and *P*<0.05). Bacterial interactions in N **(A)**, P1 **(B)**, P2 **(C)**, and P3 **(D)** are depicted. The size of the nodes is proportional to the genus abundance. Node color corresponds to the phylum classification. Edge colors indicate positive (red) and negative (blue) correlations.

Network analysis was performed to recognize interactions among genera in different groups. The top 20 genera for each group in the relative abundance ranking were selected from the four groups. Genera that met the threshold of Spearman’s correlation coefficient of ρ >0.6 and *P*<0.05 are shown in the networks (**Additional File 1: Table S9** and **Figure 4**). The modularity, node, edge, and graph density for group N were 0.30, 19, 40, and 0.30, respectively. *Streptococcus*, *Fusobacterium*, *Leptotrichia*, and *Neisseria* were present in greater relative abundance in N, compared with the other genera in the network, with *Streptococcus* and *Fusobacterium* showing negative correlation. The modularity, node, edge, and graph densities were 0.38, 19, 29, and 0.17 for P1; 0.56, 18, 25, and 0.163 for P2; and 0.63, 17, 15, and 0.11 for P3. In P1, P2, and P3, *Leptotrichia*, *Fusobacterium*, and *Prevotella* were present in greater relative abundance, compared with the other genera in the networks, and *Leptotrichia* was negatively correlated with *Fusobacterium* and *Prevotella*. These analyses suggest that group N may have a more complex network topology, and the network topology of N differs from that of the three pregnancy groups (**Additional File 1: Table S9, Additional File 2: Figure S4**).

### Predicted metabolic functions of supragingival microbiota

**Figure 5:**
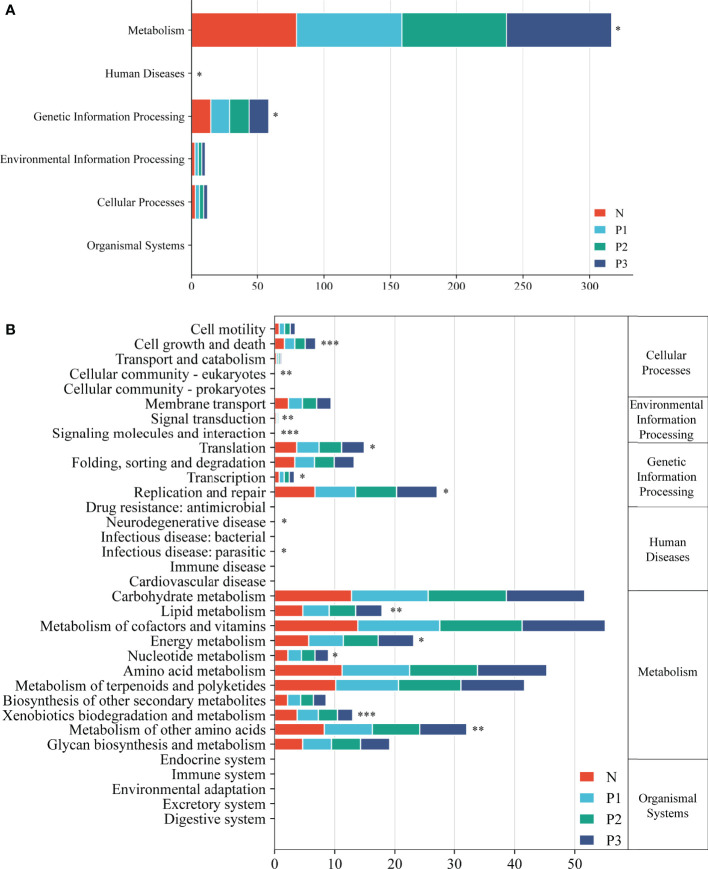
Level 1 **(A)** and level 2 **(B)** KEGG pathways in the four groups. Asterisks indicate significant differences; * *P*<0.05, ** *P*<0.01, *** *P*<0.001.

Functional pathways for the bacterial communities of N, P1, P2, and P3 were predicted using PICRUSt2 (**Additional File 1: Table S10**). Level 1 functional pathways that differed significantly among the four groups included metabolism, genetic information processing, and human diseases. The abundance of predicted functions related to metabolic pathways and genetic information processing was high in all four groups, accounting for 93.93%, 93.73%, 93.63%, and 93.76% of all level 1 predicted functions in N, P1, P2, and P3 groups, respectively (**Figure 5A**). Level 2 functional pathways that differed significantly among the four groups included metabolism of other amino acids, energy metabolism, lipid metabolism, xenobiotics biodegradation and metabolism, nucleotide metabolism, replication and repair, translation, and cell growth and death. The abundance of predicted functions related to metabolism of cofactors and vitamins, carbohydrate metabolism, amino acid metabolism, and metabolism of terpenoids and polyketides was high in all four groups, accounting for 48.01%, 48.28%, 48.51%, and 48.94% of all level 2 predicted functions in N, P1, P2, and P3 groups, respectively. (**Figure 5B**). PICRUSt2 predicted 172 level 3 functional pathways by comparison with KEGG immediate relatives (**Additional File 1: Table S10, Additional File 2: Figure S4**).

**Figure 6:**
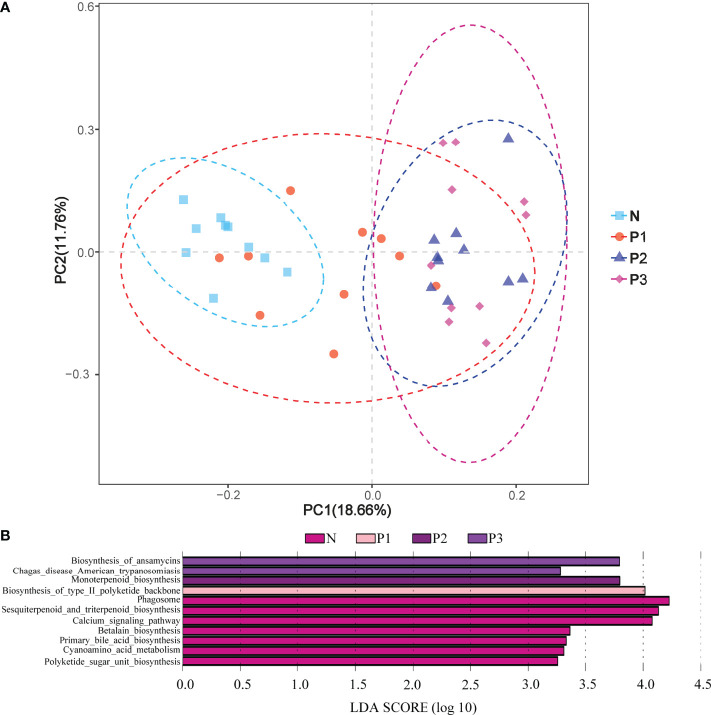
**(A)** PCA plots to assess beta diversity among the KEGG level 3 predicted microbial metabolic functions in the four studied groups. **(B)** Linear discriminant analysis (LDA) of the KEGG level 3 predicted microbial metabolic functions in the four groups. The length of the histogram represents the LDA score.

PCA demonstrated that most of the N and P1 predictive function pathways clustered together, and that the P2 and P3 predictive function pathways clustered together (**Figure 6A**). Changes in oral microbial predictive functional pathways occurred during mid- and late-pregnancy, and these changes may be related to pregnancy. Data for predicted functional pathways were converted to relative abundance and differences between groups are shown in **Figure 6B**. LEfSe analysis showed that 11 pathways played a discriminatory role in the four studied groups (**Figure 6B**). Functional pathways in the bacterial community of N that were clearly distinguished from those of the other groups included polyketide sugar unit biosynthesis, cyanoamino acid metabolism, primary bile acid biosynthesis, and betalain biosynthesis. The functional pathway that clearly distinguishes the P1 bacterial community from those of the other three groups is the biosynthesis of type II polyketide backbone. The functional pathway that clearly distinguishes P2 from the other groups is monoterpenoid biosynthesis, and the functional pathways that clearly distinguish the bacterial community of P3 from those of the other three groups include chagas disease (American trypanosomiasis) and biosynthesis of ansamycins.

## Discussion

Adverse pregnancy outcomes affect more than 20% of newborns worldwide each year (Ye and Kapila, 2021), imposing a heavy economic burden on families and society (Rogers and Velten, 2011; Chen et al., 2012). However, half of the causes are still unknown (Ye and Kapila, 2021). Intrauterine infections play a major role in adverse pregnancy outcomes. In addition to upstream infection by bacteria from the lower genital tract, intrauterine placental bacterial infections may also arise from bloodstream infection by oral bacteria (Han Y and Wang, 2013; Vornhagen et al., 2016). Therefore, studies of the oral microbiome during pregnancy are necessary to better predict and intervene in adverse pregnancy outcomes.

Advances in sequencing technology are rapidly changing the experimental landscape of microbial ecology (Kozich et al., 2013). High-throughput sequencing technologies provide an efficient and effective method for studying the composition and structure of bacterial communities associated with health and disease. The current study was conducted using Illumina MiSeq sequencing, and the supragingival plaque microbiota of pregnant and non-pregnant women exhibited significant differences in community structure and composition.

The groups of subjects in this study were not statistically different in terms of age, race, and oral status characteristics (*P*>0.05). However, BMI was higher in the second and third trimester groups compared with the non-pregnant control group (Kruskal–Wallis test, *P*<0.001). Currently, there is no agreement on the effect of elevated BMI on oral microbiology without reaching obesity levels. One study showed higher numbers of red complexes (*Porphyromonas gingivalis*, *Treponema denticola*, and *Tannellera forsythia*) in non-diabetic patients with a high BMI or waist circumference (Matsushita et al., 2015). In contrast, another study showed no statistical effect on the composition of the salivary bacterial profile based on the extreme degree of fractional stratification of parameters such as age, sex, alcohol consumption, BMI, and dietary intake (Belstrøm et al., 2014). Consequently, the effect of BMI on the results of the present study is unclear. Follow-up studies should aim to select women with similar BMIs to exclude the possible effects of BMI on the results.

Both Simpson’s index and Shannon’s index are indicators of microbial diversity (Hill et al., 2003; Kim et al., 2017). However, in the present study, these two diversity indices reflected inconsistent results. The reason for this contradictory result may be that these two diversity indices were influenced by species evenness (Hill et al., 2003; Kim et al., 2017). Most studies only use the Shannon index as a community diversity indicator, thus the following discussion focuses on the trend of the Shannon index. Previous studies have mostly reported an increase in oral microbial diversity in pregnant women compared with non-pregnant women (Fujiwara et al., 2017; Lin et al., 2018; Balan et al., 2021). Among these studies, Balan et al. found significantly higher alpha diversity in the subgingival plaque microbial community of a pregnant group compared with that of a non-pregnant group (Balan et al., 2021). Fujiwara et al. concluded that the total number of culturable microorganisms in subgingival plaque was substantially higher in early pregnancy compared with that in non-pregnant women (Fujiwara et al., 2017). These two studies used subgingival plaque samples, while the present study used supragingival plaque samples. However, although the samples were different, we demonstrated from different perspectives that oral microbial diversity changed during pregnancy. Lin et al. found that supragingival plaque microbial diversity was higher in pregnancy compared with that in non-pregnancy, with the Shannon index significantly higher in late pregnancy compared with that in the non-pregnancy group (Lin et al., 2018). These results have some similarity with the results of the present study. The samples used in that study were the same as those in the present study, both being supragingival plaque. The changes in Shannon index were essentially the same in both studies, both showing a higher oral microbial diversity in pregnancy compared with that in non-pregnancy, except that it was the late pregnancy Shannon index that was significantly higher in the study of Lin et al., whereas in the present study, it was the mid-pregnancy Shannon index that was significantly higher compared with that the non-pregnancy group (**Table 2** and **Figure 1B**). Our results are consistent with those of previous studies, which indicate a trend toward increased oral microbial diversity during pregnancy. This elevated trend is also congruent with the hormonal surge of progesterone and estradiol during pregnancy (Wu et al., 2015). However, a few studies found no significant difference in oral bacterial diversity between pregnant and non-pregnant groups. Machado et al. showed that the bacteria most commonly associated with periodontal disease did not differ in subgingival plaque between pregnant and non-pregnant women (Machado et al., 2012). Differences in samples and study methods might explain the difference in results from Machado et al. compared with those of both the present study and other studies. Since the current findings are from studies with small sample sizes, the results need to be validated in future studies with large sample sizes.

The composition of the microbiome is closely related to the disease state (Willis and Gabaldón, 2020). Significant increases in circulating levels of estrogen and progesterone are thought to have a significant effect on the periodontium throughout pregnancy (Wu et al., 2015). Therefore, it can be hypothesized that pregnancy may create a nutritional environment more favorable for certain sensitive microbial strains. A total of 15 phyla, 32 classes, 53 orders, 98 families, 194 genera, and 503 species were detected in all specimens. The predominant phyla were Fusobacteria, Bacteroidetes, Firmicutes, and Proteobacteria, which is similar to the findings of previous studies (Dewhirst et al., 2010; Lin et al., 2018). The current study showed that, at the phylum level, most of the prevalent bacteria were essentially the same in the four groups, but different relative abundances were observed among groups. At the genus level, the dominant genera differed slightly between the pregnant and non-pregnant groups. *Leptotrichia* was the most abundant genus in the pregnancy group, followed by *Fusobacterium*, *Prevotella*, and *Streptococcus*, together accounting for 50.82%, 51.85%, and 53.22% of the total OTUs in P1, P2, and P3, respectively. *Streptococcus* was the most abundant genus in the non-pregnancy group, followed by *Fusobacterium*, *Leptotrichia*, and *Neisseria*, which together accounted for 45.00% of the total OTUs in N group. As shown in the community structure bar charts and Sankey plot, the pregnant and non-pregnant groups exhibited different community structures, suggesting that pregnancy status may significantly affect bacterial composition.

In the current study, LEfSe analysis was used to identify bacterial species in each group that were significantly different from those of other groups (**Additional File 1: Table S8** and **Figures 2C, D**). The species in group N that differed significantly from those of other groups were mainly concentrated in the genus *Neisseria*. The species in group P1 that differed significantly from those in the other groups were mainly concentrated in the genus *Tannerella*. The species in groups P2 and P3 that differed significantly from those in the other groups were concentrated in the genus *Leptotrichia*. Species of the genus *Neisseria* usually colonize the nasopharynx without causing any significant pathological changes and can be considered a normal part of the respiratory microbiome, causing serious disease only when meningococci leave their usual ecological niche (Seifert, 2019). *Tannerella forsythia* is a periodontopathogenic bacterium that, together with *Porphyromonas gingivalis* and *Treponema denticola*, constitutes the “red complex”, which is a multibacterial pathogenic complex in periodontitis (Mysak et al., 2014). *Tannerella forsythia* is also one of the species most associated with halitosis and has even been associated with some systemic diseases, such as Alzheimer’s disease (Singhrao et al., 2015) and esophageal cancer (Peters et al., 2017). Members of the genus *Leptotrichia* are non-motile pathogenic bacteria found predominantly in the oral cavity and other parts of the body (Eribe and Olsen, 2017; Hou et al., 2018). All species of the genus *Leptotrichia* ferment carbohydrates and produce lactic acid, which may be associated with dental caries (Eribe and Olsen, 2017; Hou et al., 2018). It follows that pathogenic microorganisms associated with periodontitis, dental caries, and systemic diseases may increase in the mouth of healthy individuals as pregnancy progresses. This suggests that we must pay attention to oral hygiene care during pregnancy.

Network analysis was used to explore the potential relevance of the supragingival plaque microbiota detected in the current study. The modularity, node, edge, and graph density of group N were 0.30, 19, 40, and 0.30, respectively. *Streptococcus*, *Fusobacterium*, *Leptotrichia*, and *Neisseria* were present greater relative abundance in N, compared with other genera in the network, with *Streptococcus* and *Fusobacterium* showing negative correlation. This is consistent with the results of a previous study (Lin et al., 2018). The non-pregnant group showed more complex and convergent relationships among bacterial genera compared with those of the pregnant groups, suggesting that some microbial relationships may be disrupted and lead to dysbiosis of oral ecology during pregnancy.

The functional pathways of the oral microbial community were also predicted in the current study. PCA analysis showed that most predicted functional pathways of groups N and P1 clustered together, while those of P2 and P3 clustered together (**Figure 3A**). This indicates that changes in oral microbial predictive functional pathways occurred during mid- and late-pregnancy and that these changes may be related to pregnancy. LEfSe analysis also identified 11 functional pathways that were significantly different in the four groups. However, given the limitations of PICRUSt2 (Zhang et al., 2022), the analysis of predicted oral microbiota function only provided some preliminary results. Functional changes in the oral microbiota should be confirmed by combining metabolomics and macrogenomics in the future.

This study provides a preliminary assessment of the supragingival plaque microbiota and its relationship to healthy pregnant women. However, there are limitations to the study. Firstly, experimental procedures are needed to determine how pregnancy and sex hormones affect the microorganisms involved. In addition, the number of study subjects in each group was limited and the samples from the three trimesters were not from the same group of women. In future studies, the number of study subjects should be increased and oral samples should be collected from the same group of women in all three trimesters to improve the credibility of the conclusions.

## Conclusion

This study characterized the microbiota of supragingival plaque in pregnant and non-pregnant women and improved understanding of the ecological and microbial shifts associated with pregnancy. Findings from the study suggest that pregnancy has a potential role in shaping the supragingival plaque microbiota and that physiological changes during pregnancy may transform supragingival plaque into entities that may cause harm, which may be a risk factor for maternal health.

## Data availability statement

The datasets presented in this study can be found in online repositories. The names of the repository/repositories and accession number(s) can be found in the article/**Supplementary Material**.

## Ethics statement

The studies involving human participants were reviewed and approved by Research and Ethics Committee of the First Affiliated Hospital of Xinjiang Medical University. The patients/participants provided their written informed consent to participate in this study.

## Author contributions

Conceived and designed the study: JZ, YZ, and ZW; Participated in investigation: YZ and WF; Performed formal analysis: YZ, ZW, and WF; Collected the resources: YZ, LL, XW, and WF; Curated the data: YZ, LL, XW, and WF; Wrote the manuscript: YZ and LL; Supervised the study: JZ, YZ, and ZW. All authors read and approved the final version of the manuscript.

## Acknowledgments

This work was financially supported by the Natural Science Foundation of Xinjiang Uygur Autonomous Region (No. 2018D01C183), Xinjiang Uygur Autonomous Region Postgraduate Scientific Research Innovation Project (No. XJ2022G164) and the National Natural Science Foundation of China (No. 81760194). We appreciate all pregnant and non-pregnant individuals for participating in the research.

## Conflict of interest

The authors declare that the research was conducted in the absence of any commercial or financial relationships that could be construed as a potential conflict of interest.

## Publisher’s note

All claims expressed in this article are solely those of the authors and do not necessarily represent those of their affiliated organizations, or those of the publisher, the editors and the reviewers. Any product that may be evaluated in this article, or claim that may be made by its manufacturer, is not guaranteed or endorsed by the publisher.
