# Supplementary material for: Characterizing the supragingival microbiome of healthy pregnant women
Source: Front Cell Infect Microbiol. 2022 Nov 17;12:1016523. doi: 10.3389/fcimb.2022.1016523 (PMC9713012; doi:10.3389/fcimb.2022.1016523)
Supplement: Supplementary file 1 [file DataSheet_1.pdf]

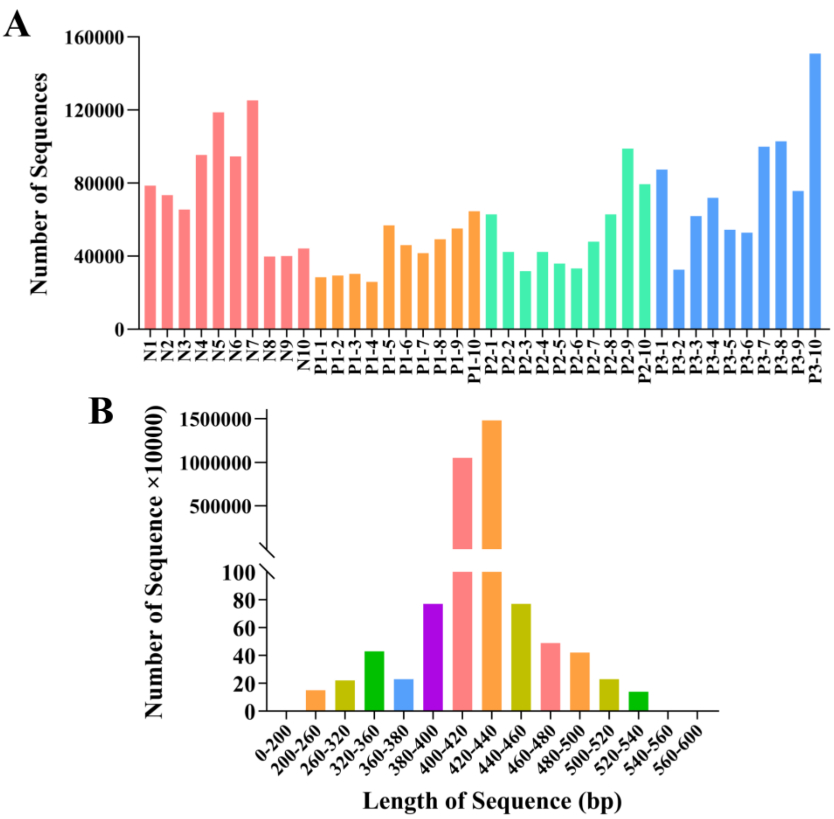

Supplementary Figure S1 **(A)** The number of sequences for each sample. The bars represent 40 individuals in four groups. **(B)** Length distribution of the qualified sequences.

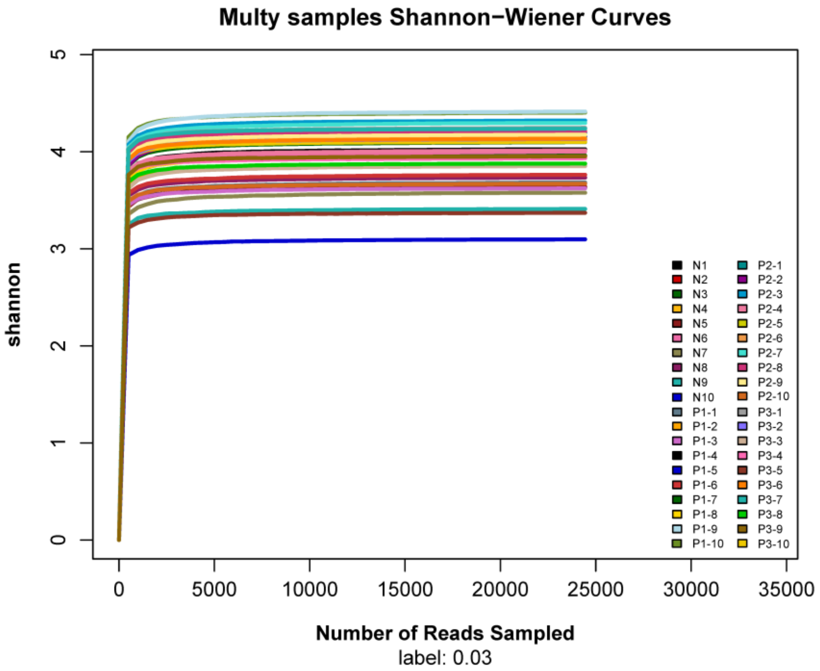

Supplementary Figure S2 Shannon-Wiener curves of each sample. Shannon-Wiener curves were all calculated at the 97% similarity level with pyrosequencing data in microbiota from four groups.

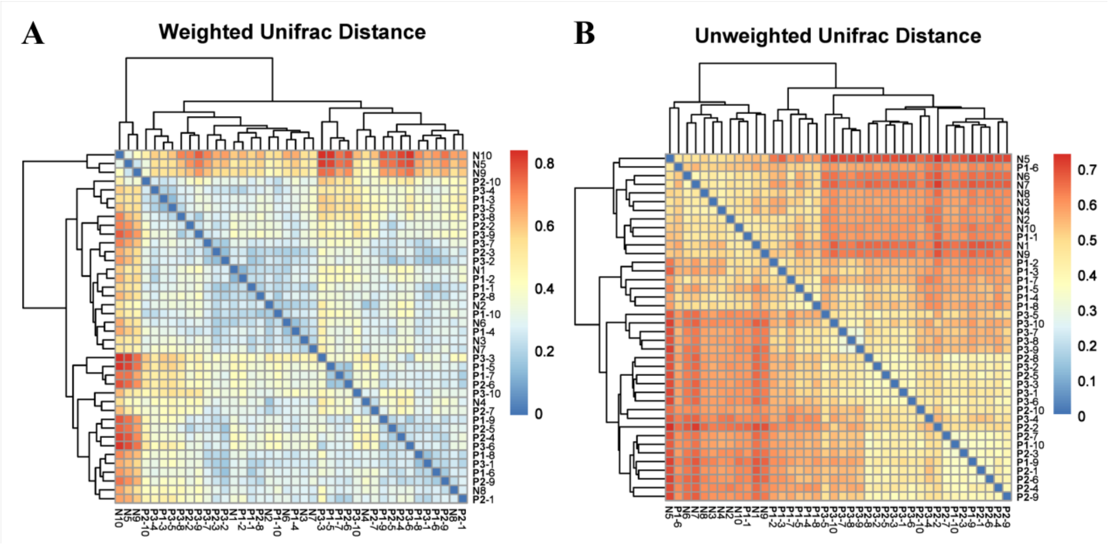

Supplementary Figure S3 **(A, B)** Heatmap based distance matrix of beta diversity analysis using weighted uniFrac **(A)** and unweighted uniFrac **(B)**. Rows and columns represent all the 40 samples, the similarity represented by the values in the heatmap and the lower number represents greater similarity in bacterial microbiota between samples in the heatmap.

A

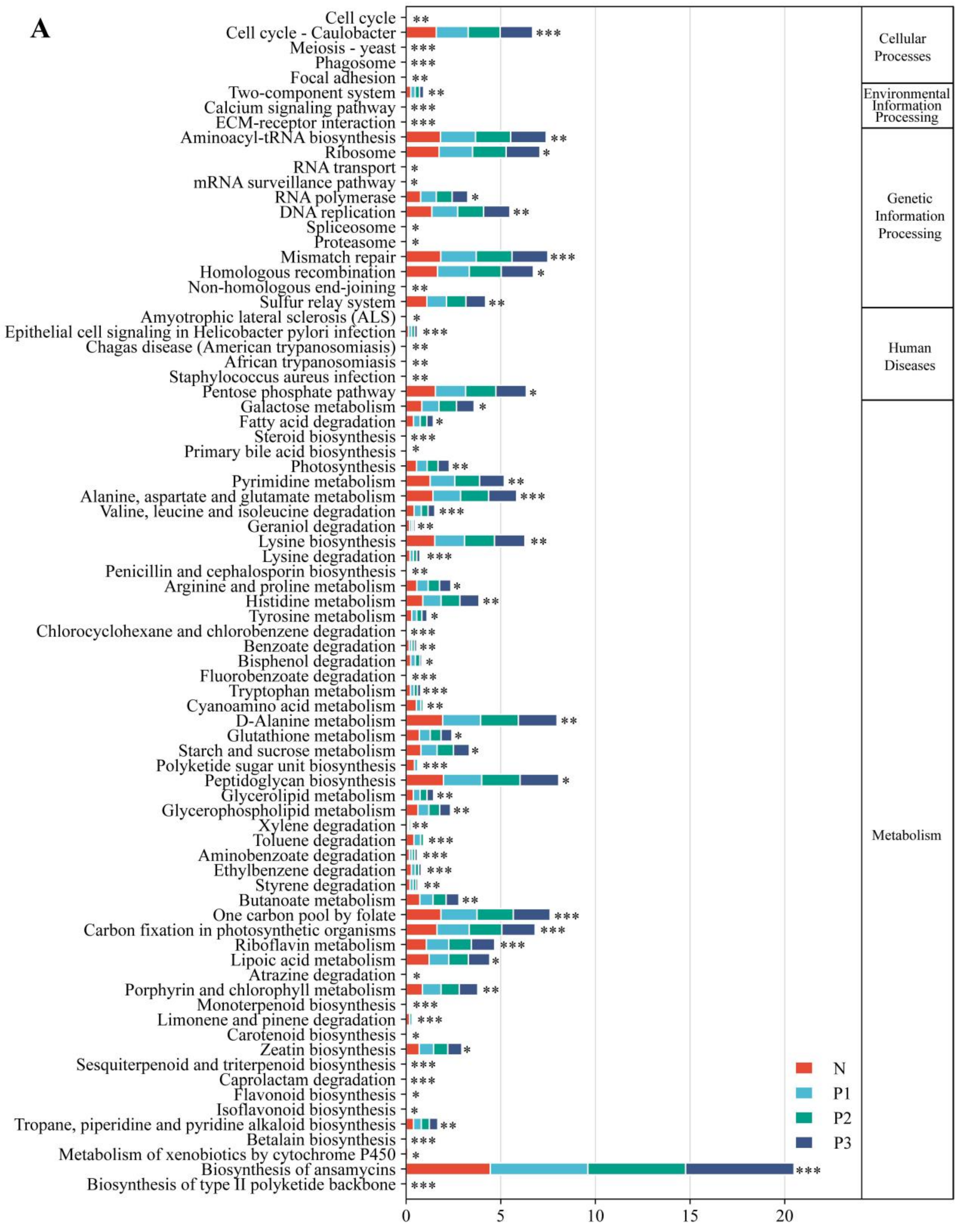
Cell cycle
Cell cycle - Caulobacter
Meiosis - yeast
Phagosome
Focal adhesion
Two-component system
Calcium signaling pathway
ECM-receptor interaction
Aminoacyl-tRNA biosynthesis
Ribosome
RNA transport
mRNA surveillance pathway
RNA polymerase
DNA replication
Spliceosome
Proteasome
Mismatch repair
Homologous recombination
Non-homologous end-joining
Sulfur relay system
Amyotrophic lateral sclerosis (ALS)
Epithelial cell signaling in Helicobacter pylori infection
Chagas disease (American trypanosomiasis)
African trypanosomiasis
Staphylococcus aureus infection
Pentose phosphate pathway
Galactose metabolism
Fatty acid degradation
Steroid biosynthesis
Primary bile acid biosynthesis
Photosynthesis
Pyrimidine metabolism
Alanine, aspartate and glutamate metabolism
Valine, leucine and isoleucine degradation
Geraniol degradation
Lysine biosynthesis
Lysine degradation
Penicillin and cephalosporin biosynthesis
Arginine and proline metabolism
Histidine metabolism
Tyrosine metabolism
Chlorocyclohexane and chlorobenzene degradation
Benzoate degradation
Bisphenol degradation
Fluorobenzoate degradation
Tryptophan metabolism
Cyanoamino acid metabolism
D-Alanine metabolism
Glutathione metabolism
Starch and sucrose metabolism
Polyketide sugar unit biosynthesis
Peptidoglycan biosynthesis
Glycerolipid metabolism
Glycerophospholipid metabolism
Xylene degradation
Toluene degradation
Aminobenzoate degradation
Ethylbenzene degradation
Styrene degradation
Butanoate metabolism
One carbon pool by folate
Carbon fixation in photosynthetic organisms
Riboflavin metabolism
Lipoic acid metabolism
Atrazine degradation
Porphyrin and chlorophyll metabolism
Monoterpenoid biosynthesis
Limonene and pinene degradation
Carotenoid biosynthesis
Zeatin biosynthesis
Sesquiterpenoid and triterpenoid biosynthesis
Caprolactam degradation
Flavonoid biosynthesis
Isoflavonoid biosynthesis
Tropane, piperidine and pyridine alkaloid biosynthesis
Betalain biosynthesis
Metabolism of xenobiotics by cytochrome P450
Biosynthesis of ansamycins
Biosynthesis of type II polyketide backbone
Cellular Processes
Environmental Information Processing
Genetic Information Processing
Human Diseases
Metabolism
N
P1
P2
P3
0
5
10
15
20

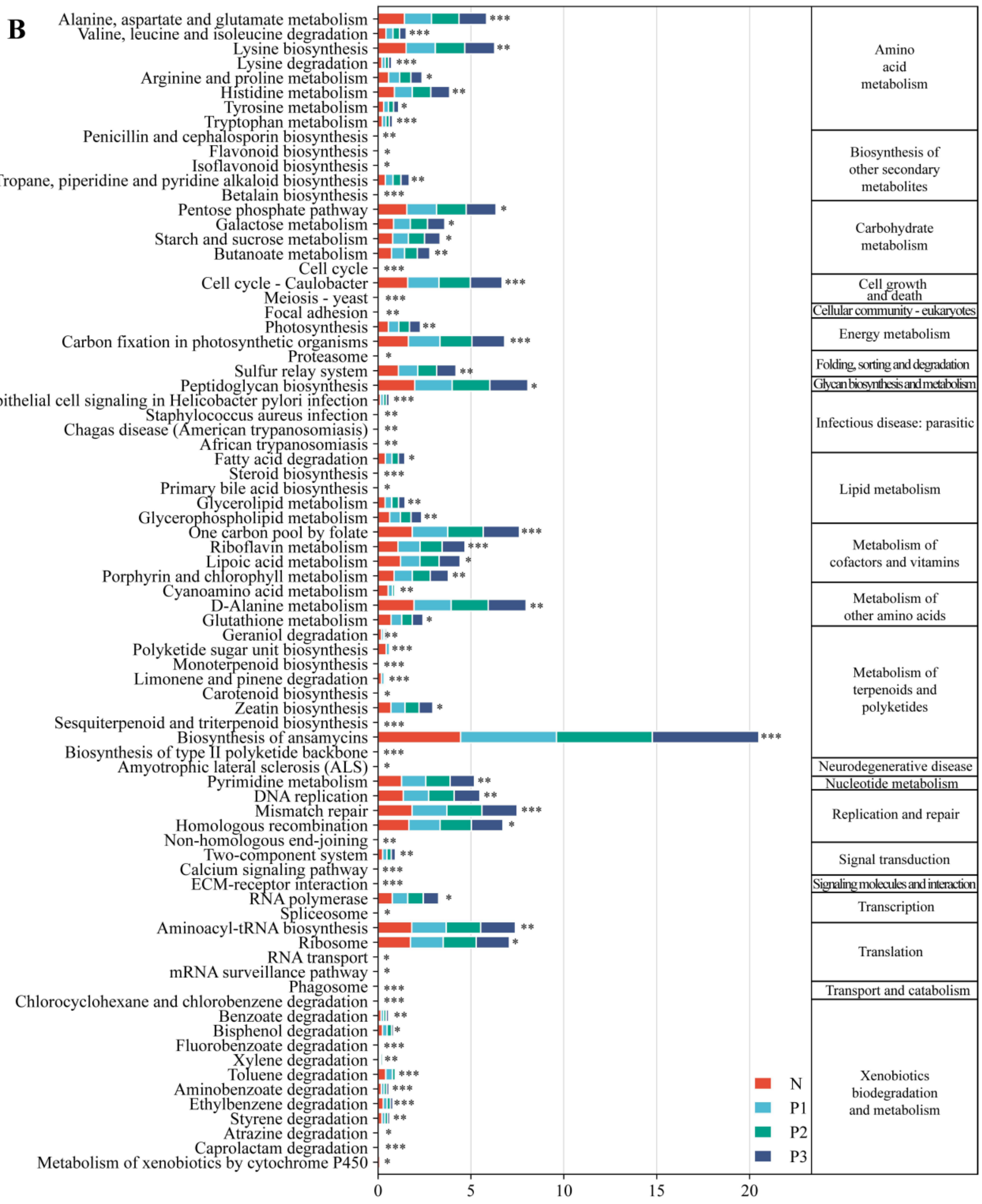

Supplementary Figure S4 **(A)**The level 1 and level 2 KEGG pathways in four groups. **(B)**The level 1 and level 3 KEGG pathways in four groups.
